# Microbial and metabolomic remodeling by a formula of Sichuan dark tea improves hyperlipidemia in apoE-deficient mice

**DOI:** 10.1371/journal.pone.0219010

**Published:** 2019-07-03

**Authors:** Lingzhi Li, Min Shi, Stephen Salerno, Minghai Tang, Fan Guo, Jing Liu, Yanhuan Feng, Martina Fu, Qinwan Huang, Liang Ma, Yi Li, Ping Fu

**Affiliations:** 1 Division of Nephrology and National Clinical Research Center for Geriatrics, Kidney Research Institute, West China Hospital of Sichuan University, Chengdu, China; 2 Department of Biostatistics, School of Public Health, University of Michigan, Ann Arbor, Michigan, United States of America; 3 College of Pharmacy, Chengdu University of Traditional Chinese Medicine, Chengdu, China; University of Hawai'i at Manoa College of Tropical Agriculture and Human Resources, UNITED STATES

## Abstract

Medicine-food homology is a long-standing concept in traditional Chinese medicine. YiNianKangBao (YNKB) tea is a medicine-food formulation based on Sichuan dark tea (Ya’an Tibetan tea), which is traditionally used for its lipid-lowering properties. In this study, we evaluated the effects of YNKB on dyslipidemia and investigated the mechanism underlying its correlation with gut microbiota and serum metabolite regulation. Wild-type mice were fed a normal diet as a control. Male ApoE-/- mice were randomly divided into three high-fat diet (HFD) groups, a model group, and two treated groups (100, 400 mg/kg/d for low, high-dose), and fed by gavage for 12 weeks. Serum lipid levels, composition of gut microbiota, and serum metabolites were then analyzed before treatment with YNKB. We extracted the ingredients of YNKB in boiled water for one hour. YNKB supplementation at a high dose of 400 mg/kg/day reduced bodyweight gains (relative epididymal fat pad and liver weight), and markedly attenuated serum lipid profiles and atherosclerosis index, with no significant differences present between the low-dose treatment and HFD groups. Gut microbiota and serum metabolic analysis indicated that significant differences were observed between normal, HFD, and YNKB treatment groups. These differences in gut microbiota exhibited strong correlations with dyslipidemia-related indexes and serum metabolite levels. Oral administration of high-dose YNKB also showed significant lipid-lowering activity against hyperlipidemia in apoE-deficient mice, which might be associated with composition alterations of the gut microbiota and changes in serum metabolite abundances. These findings highlight that YNKB as a medicine-food formulation derived from Sichuan dark tea could prevent dyslipidemia and improve the understanding of its mechanisms and the pharmacological rationale for preventive use.

## Introduction

Hyperlipidemia is a major risk factor contributing to atherosclerosis and cardiovascular disease (CVD). It is characterized by abnormal plasma lipid levels, including increased triglyceride (TG), total cholesterol (TC) and low-density lipoprotein cholesterol (LDL-c), and decreased high-density lipoprotein cholesterol (HDL-c) [1, 2]. Regulating lipid metabolism disorders is a potential approach to slowing or preventing the development of CVD. Although medications such as fibrates and statins are effective in the treatment of lipid metabolism dysfunction, their use is limited by individual differences in hyperlipidemia patients, drug dependence, and potential adverse effects including liver and kidney dysfunction, myopathy, and rhabdomyolysis [3–5].

Traditional herbal remedies with multi-functional nutrients, known as “Medicine-food homology” or “Affinal Drug and Diet,” have evolved from ancient healing systems and have enjoyed a remarkable resurrection in recent years [6, 7]. Sichuan dark tea, one of China’s dark tea representatives, is a popular medicine-food beverage for ethnic groups living in the regions of Sichuan and Tibet. It is fermented from the rough old leaves of *Camellia sinensis* with various microorganisms. Recently, considerable studies have focused on the pharmacological activity of Chinese dark tea, which has been traditionally used in drug formulations or as food for the prevention and treatment of hyperlipidemia, obesity, and diabetes [8–11].

In accordance with the theory of traditional Chinese medicine (TCM) and guidelines set by the Chinese Food and Drug Administration (CFDA) surrounding the use of foods for special medical purpose, YiNianKangBao (YNKB), as a homology of medicine and food based on Sichuan dark tea, was developed and used on the market for the preventive treatment of dyslipidemia. Specifically, sixteen traditional Chinese herbs (S1 Table), which have been reported to alleviate hyperlipidemia, atherosclerosis, and cardiovascular diseases [12–16], were formulated in a mixture with Sichuan dark tea for optimal compatibility. However, the anti-hyperlipidemic activity of YNKB and its potential mechanisms are unclear. Hence, in this study, the water extract of medicine-food YNKB was prepared and first evaluated in high-fat diet (HFD) induced apoE-deficient mice. The potential lipid-lowering function of YNKB involved in the modulation of gut microbiota composition and serum metabolite levels was also investigated.

## Materials and methods

### Preparation of medicine-food YNKB extract

YNKB, produced by the Chengdu YiNianKangBao Biological Technology Co., Ltd. (Chengdu, China), consists mainly of Sichuan dark tea, *Nelumbo nucifera* Gaertn., *Crataegus pinnatifida* Bge, *Lycium barbarum* L., and *Morus alba* L. (see S1 Table for the full display of ingredients). These components were accredited according to the Pharmacopoeia of the People’s Republic of China (2015 edition). After being fully mixed, the medicine-food ingredients were boiled twice with hot water (1L × 2), followed by filtration processes. The filtered solution containing the extract was then evaporated as YNKB for the in vivo experiment. High-performance liquid chromatography-mass spectrometry (HPLC-MS) analysis of YNKB tea is shown in S1 Fig. As the recommended dose of YNKB tea for human was 2g per day from the instruction, we converted the dose from human to mouse as a high-dose of 400mg/kg/d according to the published article [17]. Additionally, from other articles, 100mg/kg/d herbal administration showed significant antihyperlipidemic effects [18], which suggested to us to add 100mg/kg/d as the low-dose group.

### Experimental design

Seven male wild-type mice and 20 male ApoE-/- mice were obtained from the Animal Laboratory Center of Sichuan University (Chengdu, China) at 10 weeks of age. The mice were housed in a room under a 12:12-hour light-dark cycle with free access to food pellets and water. After two weeks of acclimatization with the standard chow diet, the ApoE-/- mice were then randomly assigned into three groups and fed with a 40% HFD (D12451, Research diet, New Brunswick, NJ, USA) and 0.9% saline solution for 12 weeks by gavage. The three ApoE-/- mice groups were randomized to receive just the HFD (HFD group), the HFD and a low-dose of YNKB (100 mg/kg/day; HFD+L group), or the HFD and a high-dose of YNKB (400 mg/kg/day; HFD+H group). The wild-type mice continued to be administered a standard chow diet as control (N group; D12450B, Research diet, New Brunswick, NJ, USA). See S2 Table for full details. The body weights of the mice and food intake were measured every week. After 12 weeks, all mice were euthanized by applying pentobarbital through intraperitoneal injection and samples were collected. The epididymal fat pad, liver, kidney, heart, aorta and ileum content were excised, weighed, and frozen in liquid N_2_ or stored in 10% neutral formalin. A viscera index (including relative epididymal fat pad weights and relative liver weight) was calculated using the formula: organ weight/body weight (mg/g). The protocol was approved by the animal ethics committee of Sichuan University (No. 2017080A).

### Measurement of blood biochemical indices

Serum levels of TC, TG, HDL, aspartate transaminase (AST) and alanine transaminase (ALT) were determined using a kit from BioSino Biotechnology & Science Inc. (Beijing, China).

### Histopathological analysis of the liver

Freshly isolated livers were fixed in 10% formalin for 24 hours prior to histopathological evaluation. After being rinsed under running water, the tissues were processed in a paraffin automatic processor using a programmed cascade. Paraffin-embedded liver tissues were dissected (4μm thick) and stained with hematoxylin and eosin. After staining, histopathological features were assessed under a microscope. One photograph per sample was obtained using both a 200x and 400x optical microscope.

### Determination of lipid levels in liver tissue

Hepatic TC and TG contents were determined using commercially available kits from BioSino Biotechnology & Science Inc. (Beijing, China).

### 16S rRNA gene sequencing

Microbial DNA was extracted from ileum content samples using the E.Z.N.A. DNA Kit (Omega Bio-tek, Norcross, GA, U.S.) according to the standard protocol. The V3-V4 hypervariable regions of the bacteria 16S rRNA gene were amplified with primers 338F (5’- ACTCCTACGGGAGGCAGCAG-3’) and 806R (5’-GGACTACHVGGGTWTCTAAT-3’) by thermocycler PCR. Purified amplicons were pooled and paired-end sequenced (2 × 300) on an Illumina MiSeq platform (Illumina Inc., San Diego, CA, USA) according to the standard protocols. All raw reads were screened according to barcode and primer sequences, using Quantitative Insights Into Microbial Ecology (QIIME, version 1.17), with the following criteria. (1) The reads were truncated at any site receiving an average quality score < 20 over a 50 bp sliding window. (2) Primers were exactly matched, allowing for 2 mismatched nucleotides, and reads containing ambiguous bases were removed. (3) Sequences with overlaps longer than 10 bp were merged according to their overlap sequence. Operational taxonomic units (OTUs) were clustered with a cut-off of 97% similarity using UPARSE (version 7.1), and UCHIME was utilized to identify and remove chimeric sequences. The raw reads were deposited into the NCBI Sequence Read Archive (SRA) database (Accession Number: SRR9028650).

### Serum metabolomic analysis

200 μL of each sample were thawed at 4°C, transferred into a centrifugal tube together with 800μl of methanol, and mixed by vortexing for 60s. After centrifuging at 12,000 rpm for 10 minutes, the supernatant in each tube was transferred to another tube. The samples were blow-dried in vacuum, dissolved in a 300μL methanol aqueous solution (4:1, 4°C), and filtered through a 0.22 μm membrane. HPLC-MS sample extracts were then obtained. 20μL of each prepared sample extraction were taken for quality control (QC), and the rest were carried forward for HPLC-MS testing. Chromatographic separation was accomplished in an Acquity UPLC system equipped with an ACQUITY UPLC BEH C18 (100×2.1 mm, 1.7 μm, Waters) column maintained at 4°C. Gradient elution of analytes was carried out with 0.1% formic acid in water (A) and 0.1% formic acid in acetonitrile (B) at a flow rate of 0.25 mL/min. Injections of 5μL of each sample were done after equilibration. An increasing linear gradient of solvent B (v/v) was used as follows: 0~1min, 2%B; 1~9.5min, 2%~50%B; 9.5~14min, 50%~98%B; 14~15min 98%B; 15~15.5min, 98%~2%B; 15.5~17min, 2%B. The ESI-MS experiments were executed on the Thermo LTQ-Orbitrap XL mass spectrometer with a spray voltage of 4.8kV and -4.5kV in positive and negative modes, respectively. Sheath gas and auxiliary gas were set at 45 and 15 arbitrary units, respectively. The voltages of the capillary and tube were 35V and 50V, and -15V and -50V in the positive and negative modes, respectively. The Orbitrap analyzer scanned over a mass range of m/z 89–1000 for the full scan at a mass resolution of 60000. Data dependent acquisition (DDA) MS/MS experiments were performed with CID scan. The normalized collision energy was 30eV. Dynamic exclusion was implemented with a repeat count of 2 and exclusion duration of 15s.

### Statistical analysis

Statistical analysis was carried out in the SPSS 22.0 software (SPSS Inc., Chicago, IL, USA) and R, version 3.3.2. Statistical comparisons were assessed with a one-way ANOVA and Student’s t-test for each paired experiment, and the Wilcoxon rank sum test was used to analyze data that did not meet the assumptions of the Student’s t-test. The beta diversity of gut microbiota was assessed by a metric multidimensional scaling method based on a projection known as principal coordinates analysis (PCoA). Each sample was mapped based on the overall microbial composition and assessed for similarities. The specific characterization of fecal microbiota to distinguish taxonomic types was analyzed by the linear discriminant analysis (LDA) effect size (LEfSe) method. Using a normalized relative abundance matrix, LEfSe performs the Kruskal-Wallis rank sum test to determine the features with significantly different abundances between assigned taxa and uses LDA to assess the effect size of each feature. Correlations between abundances of gut microbiota, hyperlipidemia-related indexes, and serum metabolites were identified using Spearman’s correlation and visualized by heatmap. The PICRUSt package was used to construct the Kyoto Encyclopedia of Genes and Genomes (KEGG) Orthology (KO) and KEGG pathway/module profile, predicting functional profiling of microbial communities using 16S rRNA marker gene sequences. The pathway set enrichment analyses were performed using Metabolanalyst (www.metabolanalyst.ca) to elucidate the metabolic pathways affected by metabolite distinctions among hyperlipidemia ApoE-/- mice and the treated groups. P-values less than 0.05 were regarded as significantly different.

## Results

### High-dose YNKB attenuated bodyweight, hyperlipidemia and fatty liver in HFD-induced ApoE-/- mice

**Fig 1:**
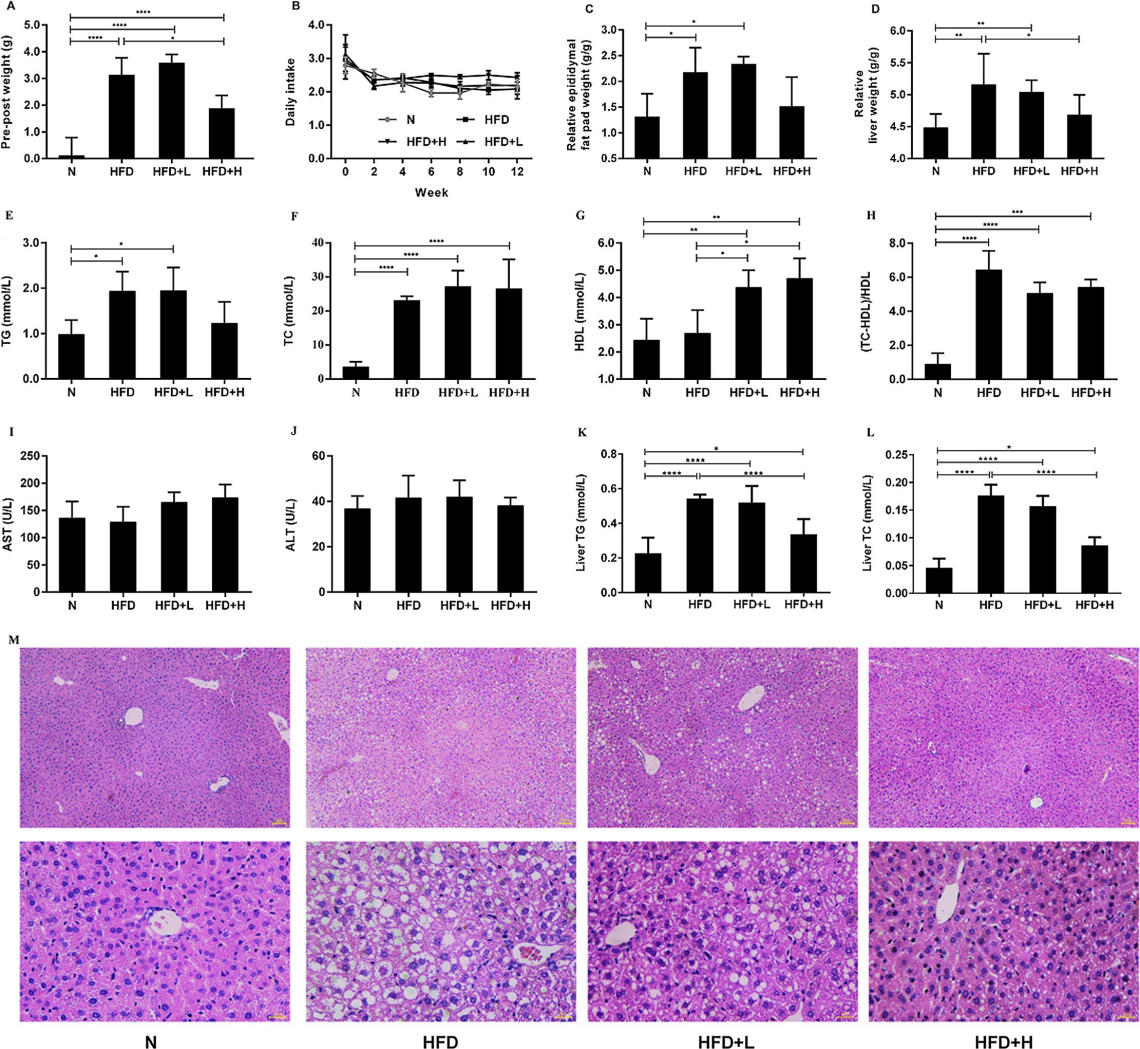
Effects of YNKB on physical features, biochemical features and histopathological findings of HFD-induced ApoE-/- mice. (A) The relative body weight gains; (B) The daily food intake (measured weekly); (C) The relative epididymal pad weights; (D) The relative liver weights of ApoE-/- mice; (E) TG; (F) TC; (G) HDL; (H) Atherosclerosis index; (I) AST and (J) ALT of ApoE-/- mice with YNKB supplementation; (K) Hepatic triglyceride and (L) cholesterol levels were determined. (M) Hepatic tissues were evaluated by HE staining. All photos are displayed under a 200x and 400x magnification. Data are presented as the mean ± S.D. *p<0.05, **p<0.01, ***p<0.001, ****p<0.0001.

After 12 weeks, the HFD group gained significantly more weight than the normal (N) group (p < 0.0001; Fig 1A). The high-dose treatment (HFD+H) group was shown to decrease bodyweight gains in the HFD-induced ApoE-/- mice (p < 0.05), while the low-dose treatment (HFD+L) group showed no significant difference when compared to the HFD group (Fig 1B). As indicated by the percentage of body weight, the epididymal fat pad and liver weighed more in the HFD group than in the N group (Fig 1C and 1D). Additionally, the relative epididymal fat pad weights in the high-dose treated HFD+H group decreased slightly, while the relative liver weight was significantly reduced when compared to the HFD group (p < 0.05).

With respect to the serum lipid indexes, the ApoE-/- mice in the HFD group developed remarkable features of hyperlipidemia, with a significant increase in the TG concentration (p < 0.05), TC concentration (p < 0.001), and atherosclerosis index (p < 0.001; Fig 1E–1H). The HFD+H group exhibited lowered TG levels and atherosclerosis index and increased HDL concentration. However, no differences in serum TC concentration were observed after 12 weeks of YNKB treatment. AST and ALT levels showed no differences among the four groups (Fig 1I and 1J). Altogether, these results suggested that the high-dose tea treatment significantly alleviated HFD-induced weight gains and hyperlipidemia.

The high-fat diet markedly increased hepatic TC and TG levels (Fig 1K and 1L). However, the increases in hepatic TC and TG were significantly attenuated by the high-dose tea administration when compared with the HFD group (p < 0.0001; Fig 1K and 1L). The high-fat diet induced lipid accumulation in hepatic tissue, as evidenced by HE staining in the HFD group, was also significantly ameliorated by tea treatment (Fig 1M).

### Transmissible microbial remodeling by hyperlipidemia and YNKB treatment

**Fig 2:**
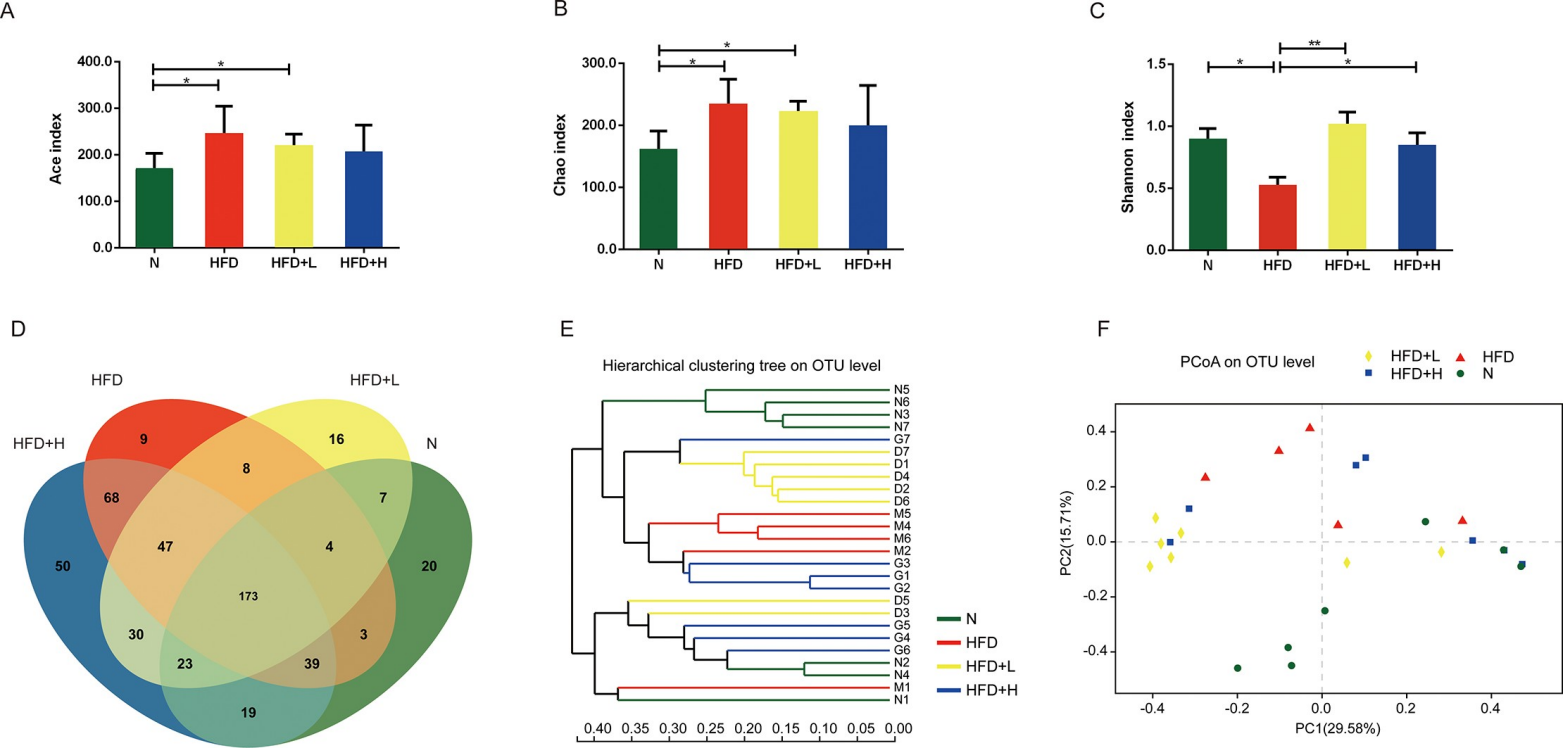
Microbiota changes in the composition of HFD-induced ApoE-/- mice by YNKB. The rarefaction curves of alpha diversity ((A) Ace, (B) Chao, or (C) Shannon index); (D) Venn diagram of the OTUs in the three groups; The beta diversity as measured by (E) hierarchical clustering tree and (F) unweighted Unifrac PCoA analysis.

Due to the deficiency of ileum content in HFD-fed ApoE-/- mice, nineteen mice were included in the microbiota analysis. A total of 514 OTUs were obtained at a 97% homology cutoff. Alpha diversity curves indicated that a significant increase in richness, measured by the Chao index, was observed in the HFD group compared to the N group at OTU level (Fig 2A and 2B). However, the treatment of YNKB decreased the richness of bacterial communities. The diversity of the microbial communities, measured by the Shannon diversity indices, was decreased in the HFD group compared to the N group, while treatment restored bacterial diversity in the HFD+L and HFD+H groups (Fig 2C). 173 OTUs were shared by all groups (Fig 2D).

The overall compositions of gut microbiota in the four groups at the phylum level were analyzed via hierarchical clustering tree and unweighted unifrac principal coordinates analysis at the OTU level (Fig 2E and 2F). The different doses of YNKB were shown to shift the overall compositions of the gut microbiota in the HFD group toward the composition of the normal diet group.

**Fig 3:**
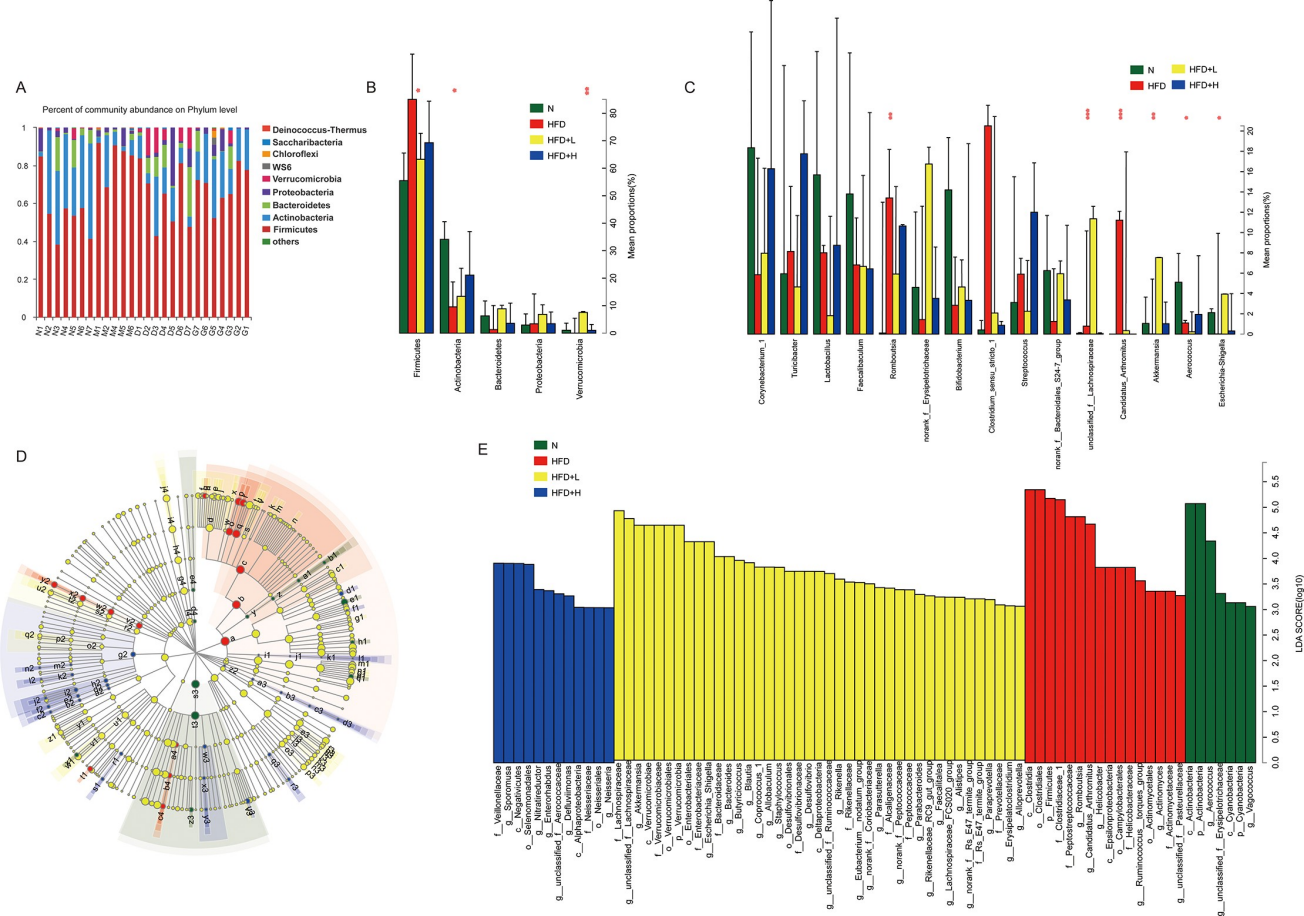
Effects of YNKB on gut microbiota relative proportion in HFD-induced ApoE-/- mic at phylum level. (A) The main microbiota and their relative proportions in different groups. Data are presented as means. The top five and fifteen microbiotas and their relative proportions in different groups at phylum level (B) and genus level (C). Differences in dominant microorganisms among groups were shown in (D) Cladogram and (E) Distribution histogram based on LDA. *p<0.05, **p<0.01 among the four groups.

Results of the taxon-based analysis revealed that the gut microbiome consisted of five dominant phyla: Firmicutes, Actinobacteria, Bacteroidetes, Proteobacteria, and Verrucomicrobia (Fig 3A). After 12 weeks of a HFD in ApoE-/- mice, widespread changes in the structure of gut microbiota were observed at the phylum level, with significantly increased proportions of Firmicutes and decreased proportions of Actinobacteria, Bacteroidetes, and Verrucomicrobia. The different doses of YNKB treatment attenuated the changes in the gut microbiota structures by decreasing proportions of Firmicutes and increasing proportions of Actinobacteria, Verrucomicrobia, and Bacteroidetes (Fig 3B).

Among the top fifteen abundant genera, the relative abundances of *Romboutsia* (p<0.01) and *Candidatus* Arthromitus (p<0.001) were significantly increased in the HFD group compared to those of the normal diet group, while *Akkermansia* (p<0.01) and *Escherichia-Shigella* (p<0.05) abundances were considerably decreased (Fig 3C, Table 1). Other genera such as *Lactobacillus* and *Bifidobacterium* also showed a trend in reduction, though without a statistic difference. The YNKB treatment, especially at the high-dose level, significantly restored the abundance of several genera such as *Candidatus* Arthromitus (p<0.05), *Akkermansia* (p<0.05) and *Escherichia-Shigella* (p<0.05) (Fig 3C, Table 1).

**Table 1:** The changes of the top fifteen genera by the treatment of YNKB.

Genus	HFD ratio (%)	N ratio (%)		HFD+L ratio (%)		HFD+H ratio (%)	
*Corynebacterium_1*	5.83±8.35	18.33±16.49	↑	7.97±11.51	↑	16.27±11.41	↑
*Turicibacter*	8.17±7.08	5.94±5.20	↓	4.63±6.41	↓	17.74±20.48	↑
*Lactobacillus*	7.97±9.68	15.69±22.36	↑	1.80±0.72	↓	8.77±12.18	↑
*Faecalibaculum*	6.74±8.89	13.77±15.42	↑	6.64±4.61	↓	6.40±13.85	↓
*Romboutsia*	13.35±8.55	0.11±0.14*	↓	5.93±4.79	↓	10.69±12.95	↓
*norank_f_Erysipelotrichaceae*	1.43±1.65	4.6±5.01	↑	16.8±11.19	↑	3.54±7.44	↑
*Bifidobacterium*	2.83±2.67	14.18±15.44	↑	4.64±4.77	↑	3.32±5.15	↑
*Clostridium_sensu_stricto_1*	20.58±19.41	0.40±0.38*	↓	2.07±2.00	↓	0.86±0.92	↓
*Streptococcus*	5.94±5.04	3.14±4.87	↓	2.26±1.58	↓	12.00±12.37	↑
*norank_f_Bacteroidales_S24-7_group*	1.24±1.27	6.27±7.37	↑	5.92±5.17	↑	3.34±5.38	↑
*unclassified_f_Lachnospiraceae*	0.77±1.21	0.082±0.079	↓	11.35±11.35*	↑	0.05±0.07	↓
*Candidatus_Arthromitus*	11.26±17.6	0*	↓	0.34±0.87*	↓	0.0005±0.001*	↓
*Akkermansia*	0.017±0.030	1.04±2.13*	↑	7.53±5.428*	↑	1.04±2.64	↑
*Aerococcus*	1.09±1.98	5.13±5.83	↑	0.22±0.24	↓	1.95±2.85	↑
*Escherichia-Shigella*	0.017±0.027	2.12±3.66*	↑	3.96±9.98*	↑	0.31±0.38*	↑

*p<0.05 versus model group.

To further identify the specific bacterial taxa associated with dyslipidemia, the compositions of the fecal microbiota for the ApoE-/- mice and healthy controls were compared using the LEfSe method. A cladogram representing the structures of the fecal microbiota and predominant bacteria in the ApoE-/- mice and healthy controls are displayed in Fig 3D. The differences in the taxa between these groups were also compared. In total, the LEfSe analysis revealed 76 discriminative features (LDA > 3, Fig 3E). Taken together, these data indicated that the ApoE-/- mice treated with YNKB had different abundance levels of certain bacteria in the gut microbiota when compared to the N and HFD groups.

### Specific microbial taxa were associated with the features of hyperlipidemia

**Fig 4:**
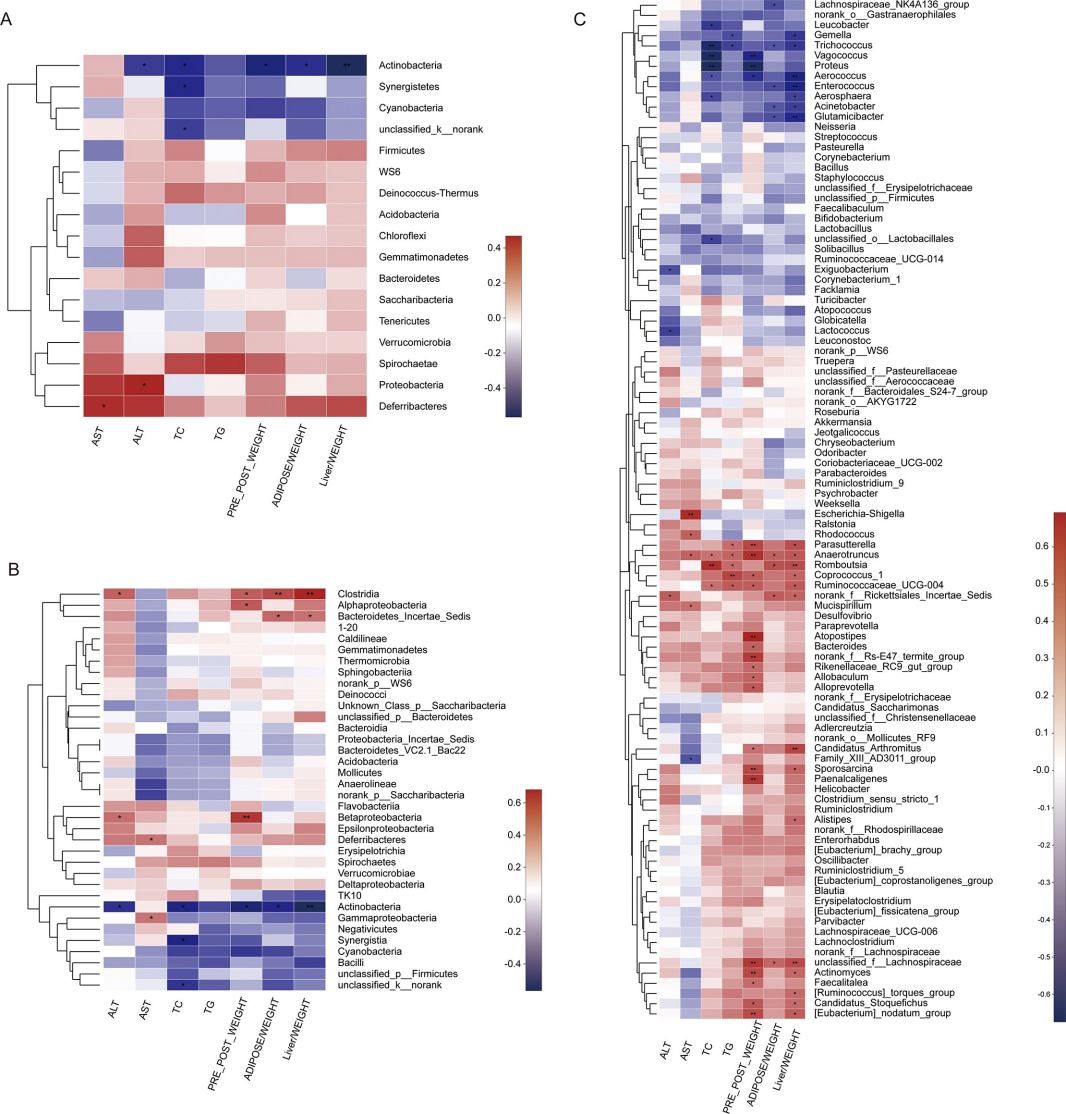
Spearman’s correlation between the identified microbiota at different levels. ((A) phylum, (B) class, and (C) genus) and the ALT, AST, TC, TG, pre-post weight, relative epidydimal adipose weight and liver weight. The color of the squares represents the R-value of Spearman’s correlation. *p<0.05, ** p <0.01, *** p<0.001.

Correlations between the shift of microbiota and clinical biomarkers were analyzed using Pearson correlation analysis. At the phylum level, Actinobacteria, Synergistetes, and cyanobacteria showed strong negative correlations with the hyperlipidemia index (Fig 4A). At the class level, Epsilonproteobacteria, Clostridia, and Alphaproteobacteria exhibited robust positive correlations with pre-post weight gains, relative epidydimal fat pat weights, and relative liver weights, while Negativicutes, Actinobacteria, cyanobacteria, TK10 and Synergistetes showed opposite relationships (Fig 4B). At the genus level, *Enterococcus*, *Glutamicibacter*, *Aerococcus*, *Vagococcus*, and *Proteus* showed negative correlations with weight gains or relative organ weights, among which *Vagococcus* and *Proteus* showed the strongest negative associations. *Trichococcus*, *Vagococcus*, unclassified_o_Lactobacillales, *Thermocirga*, and *Proteus* showed negative associations with TC or TG. However, there were more genera illustrating positive correlations with these hyperlipidemia-related indexes, including *Paresutterella*, *Atopostipes*, *Coprococcus-1*, *Ruminococcaceae-UCG-004*, unclassified_f_Lachnospiraceae, *Ruminiclostridum-5*, *Sporosarcina*, *Candidatus* Arthromitus, *Helicobacter*, *Actinomyces*, *Romboutsia*, and others. (Fig 4C). Among these genera, *Romboutsia*, unclassified_f_Lachnospiraceae, *Candidatus* Arthromitus, and *Helicobacter* were significantly increased in the HFD group and reversed by YNKB treatment (Table 1). These findings indicate that the alterations of gut microbiota induced by YNKB are associated with hyperlipidemia-related markers.

### Pronounced metabolomic shift in response to hyperlipidemia and YNKB treatment

**Fig 5:**
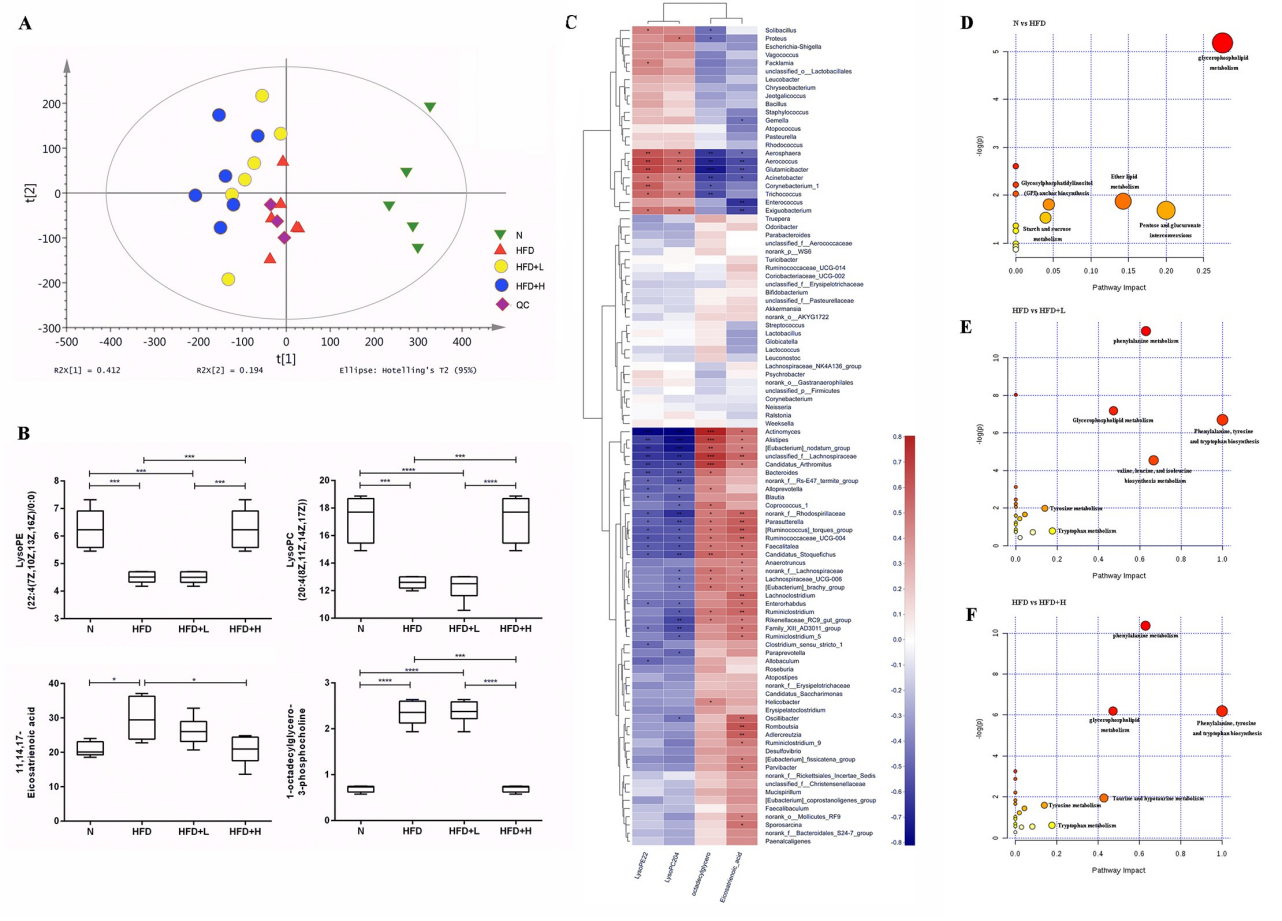
Pronounced metabolomic shift in response to hyperlipidemia and YNKB treatment. (A) PCA analysis of serum metabolites; (B) Four metabolites with differential abundance between the normal and HFD groups, restored by YNKB administration; (C) Spearman’s correlation between the identified four metabolites and microbiota at the genus level. The color of the squares represents the R-value of Spearman’s correlation. The pathway set enrichment analyses were performed using Metabolanalyst (www.metabolanalyst.ca) to elucidate the metabolic pathways affected by metabolite distinctions between the normal, HFD, and YNKB treatment groups; (D) The analysis between the N and HFD groups; (E) The analysis between the HFD and HFD+L groups; (F) The analysis between the HFD and HFD+H groups. *p<0.05, ** p <0.01, *** p<0.001.

The PCA score plots and quality control (QC) results are shown in Fig 5A. A total of 2,343 metabolites (947 in the negative ion mode, 1398 in the positive ion mode) were detected in the N, HFD, HFD+L and HFD+H groups. To investigate the global metabolite profiles, we used an FDR-corrected p-value < 0.05 as the threshold for statistical significance in the present study. Four metabolites were found to be differentially abundant in the HFD groups, with levels restored by YNKB administration. As shown in the box plots (Fig 5B), the levels of LysoPE(22:4(7Z,10Z,13Z,16Z)/0:0) and LysoPC(20:4(8Z,11Z,14Z,17Z)) were reduced significantly in the HFD samples, when compared to the normal samples. However, 11,14,17-Eicosatrienoic acid and 1-octadecylglycero-3-phosphocholine levels were increased significantly in the HFD group compared to the normal group. High-dose treatments with YNKB were shown to potentially restore these changes.

After analyzing the correlations between the four metabolites and gut microbiota at the genus level (Fig 5C), we found that the genera (*Enterococcus*, *Glutamicibacter*, *Aerococcus* and *Trichococcus*) showed negative correlations with hyperlipidemia-related parameters, had strong positive correlations with LysoPE(22:4(7Z,10Z,13Z,16Z)/0:0) and LysoPC(20:4(8Z,11Z,14Z,17Z)), and were negatively correlated with 11,14,17-Eicosatrienoic acid and 1-octadecylglycero-3-phosphocholine. However, there were more genera which illustrated positive correlations with these hyperlipidemia-related indexes (including *Paresutterella*, *Coprococcus-1*, *Ruminococcaceae-UCG-004*, unclassified_f_Lachnospiraceae, *Ruminiclostridum-5*, *Sporosarcina*, *Candidatus* Arthromitus, *Helicobacter*, *Actinomyces*, and *Romboutsia*), which also had negative relationships with LysoPE(22:4(7Z,10Z,13Z,16Z)/0:0) and LysoPC(20:4(8Z,11Z,14Z,17Z)), and positive correlation with 11,14,17-Eicosatrienoic acid and 1-octadecylglycero-3-phosphocholine.

The pathway set enrichment analyses were performed using Metabolanalyst (www.metabolanalyst.ca) to elucidate the metabolic pathways affected by metabolite distinctions between the normal, HFD, and YNKB treatment groups. The comparison between the N and HFD groups revealed pertubations of 13 networks, among which glycerophospholipid metabolism had the highest pathway impact (Fig 5D). When comparing the HFD and YNKB treatment groups, we found that low-dose YNKB treatment mainly influenced phenylalanine metabolism and valine, leucine, and isoleucine biosynthesis metabolism (Fig 5E), while a high-dose of YNKB primarily impacted phenylalanine metabolism and glycerophospholipid metabolism (Fig 5F).

## Discussion

In this study, the high-dose YNKB treatment provided significant and meaningful reductions in physical measures (pre-post body weight gains, relative epididymal fat pad weights, and relative liver weights), biochemical indexes (TC, TG, and atherosclerosis index), and hepatic lipid accumulation when compared to those of the HFD group. Additionally, the HDL level was significantly improved after YNKB treatment. Although the benefits of YNKB have not been previously reported, the lipid lowering benefits of these major components have all been described and confirmed in previous studies. *Nelumbo nucifera* Gaertn., one of the major components, was beneficial for the suppression of obesity by inhibiting the absorption of lipids and carbohydrates, accelerating lipid metabolism, and up-regulating energy expenditure [16]. Additionally, *Nelumbo nucifera* Gaertn. was shown to successfully ameliorated type 2 [19] or type 1 diabetes mellitus progress and its complications [13]. *Morus alba* L. and *Lycium barbarum*, other commonly utilized traditional Chinese herbs, were indicated to reduce the concentration of fasting glucose, TG, TC, and atherosclerotic lesion development in experimental studies [20, 21] and clinical research [22]. *Lycium barbarum* was also reported to be effective in the protection of liver and kidney tissue from damage in STZ-induced diabetic rats [23]. Additionally, the extracts from the *Morus alba* L. fruit and Hawthorn are capable of decreasing glucose production and triacylglycerol synthesis, which have antidiabetic and antihyperlipidemic benefits [12, 24, 25].

With respect to gut microbiota, several previous studies mentioned the importance of the ileum on the control of metabolic disease and bacterial translocation [26–28]. It was even found that drastic changes in the intestinal immune system occur in the ileum, rather than in the colon, in response to a fat-enriched diet, which suggested that the bacteria interacting with the host could be distributed locally rather than widely throughout the gut [26]. According to these previous studies, we choose the ileum contents to test microbiota alteration. Recent studies regarding the green and black tea polyphenols illustrated that tea could influence the structure of gut microbiota [29]. However, YNKB, as drug homologous food, has never been reported to alter microbiota composition. In this study, we found that the microbiota diversity of the HFD group decreased significantly, which was consistent with a previous study showing the lower diversity in obese patients compared to lean individuals [30, 31]. After treatment with YNKB, the decline in microbiota diversity was significantly reversed. With the highly diverse gut microbial environment, microbes will prefer to spend resources on competing and cooperating, rather than on manipulating their hosts, and hosts were indicated to be more resistant to invasion by pathogenic species [32, 33]. As to Fig 2F, we found that the HFD+L group seemed more effective in shifting the gut microbiota compositions toward the composition of the N group. This result was consistent with previous studies, which found that medium doses of resveratrol, rather than high doses, induced more obvious shifts in gut microbiota composition according to principal component analysis [34] and the highest richness was observed for the low violacein group, followed by the high violacein group and then the control group [35]. It is possible that the results might be influenced by the small sample size in these animal studies. However, we still have no evidence to explain this phenomenon. Similar to previous experiments, not only did the diversity of the microbiota change, but the structure of the microbiota in the HFD group was also converted, presenting obesity-like gut microbiota composition with increased Firmicutes and decreased Bacteroidetes and Bifidobacterium [36]. An increased abundance of Firmicutes was related to the accumulation of lipid droplets, promoting fatty acid absorption in the initiation of obesity and atherosclerosis [37–39]. Bifidobacterium and Lactobacillus, as the beneficial taxa, may contribute to the significant reduction of serum lipids and the alleviation of characteristic parameters of metabolic syndrome [40, 41]. Due to these previous studies, we hypothesize that the increasing diversity and changing composition of gut microbiota by YNKB will help to attenuate hyperlipidemia. However, the detailed mechanism remains to be further explored.

In order to determine the specific microbiota influencing hyperlipidemia, we focused our analysis at the genus level and found that concentrations of *Clostridium sensu stricto 1*, *Romboutsia*, and *Candidatus* Arthromitus increased significantly in HFD group, as compared with those of N group, while the concentrations of *Akkermansia*, and *Escherichia-Shigella* significantly decreased. After YNKB administration, the concentrations of *Akkermansia*, *Escherichia-Shigella*, and *Candidatus* Arthromitus were restored following the improvement of hyperlipidemia features. Today, growing evidence indicates that the increased intestinal abundance of *Akkermansia* protects against obesity-linked metabolic syndrome [42, 43] and contributes to the beneficial metabolic effects of the antidiabetic drug metformin [44–46] by restoring the decreased mucus layer and improving gut permeability [47]. These conclusions are consistent with our findings. *Streptococcus*, enriched by high-dose YNKB in our study, also successfully decreased serum cholesterol levels in the other hyperlipidemia rats accompanied with *Lactobacillus* [48]. However, *Escherichia-Shigella*, a genus which was once reported to be positively associated with necrotizing enterocolitis [49], was decreased markedly in our model group and was reversed by the YNKB therapy.

In addition, we found several bacterial taxa in the gut correlated with the hyperlipidemia biomarkers, including both physical and biochemical indices. We note that the proportions of *Clostridium*, *Candidatus* Arthromitus, and *Romboutsia* were positively correlated with weight gains and gains in relative organ weights. After YNKB treatment, the concentration of *Clostridium sensu stricto 1* and *Candidatus Arthromitus* dramatically dropped, characterizing a reverse in hyperlipidemia, which was consistent with these previous findings [50]. These microbiota functions might be associated with gut permeability, as *Clostridium* was reported to contribute to infection of necrotizing enterocolitis [49]. We also discovered that *Cyanobacteria*, *Vagococcus*, *Proteus*, *Aerococcus*, and *Enterococcus* showed strong negative correlations with hyperlipidemia indexes, which were reported before or newly announced [51, 52].

Data on gut microbiota perturbations associated with metabolic phenotype changes have been used to understand the possible mechanisms in the development of diseases such as obesity and hepatopathy [53, 54]. Herein, we performed serum metabolic analysis and observed a significant correlation between gut microbiota genus and serum metabolites using Spearman’s correlation analysis. We found that the serum metabolic profile of dyslipidemia ApoE-/- mice was significantly different from that of the normal controls. The concentrations of the four significantly altered metabolites, LysoPE (22:4(7Z,10Z,13Z,16Z)/0:0), LysoPC(20:4(8Z,11Z,14Z,17Z)), 11,14,17-Eicosatrienoic acid, and 1-octadecylglycero-3-phosphocholine, displayed strong correlations with several microbiota, including *Candidatus* Arthromitus and *Romboutsia*, which were also strongly connected with dyslipidemia-related indexes. Previous studies mentioned that LysoPC and LysoPE, which are structural components of animal cell membranes, are decreased in acute liver injury, acute coronary syndrome, fetal congenital heart defect, and schizophrenia [55–57] and could be identified as biomarkers for progression from early to advanced aristolochic acid nephropathy and advanced aristolochic acid nephropathy [58]. In children with substantial weight loss, LysoPC (20:4) increased significantly with increased lipolysis and a lower rate of fatty acid oxidation [59], which was consistent with our findings. Octanoylcarnitine was also reported to increase in obese adolescents when compared to non-obese adolescents [60] and change in the presence of other diseases as well [61]. These findings support the beneficial effects of these microbiota changed by YNKB and the associations of altered metabolites and gut microbiomes. Further investigations to confirm the mechanisms that link the gut microbiome and metabolic alterations are necessary in the future.

## Conclusion

The study suggests that a high-fat diet in ApoE-/- mice induces hyperlipidemia, rapid changes to crucial taxon in the gut microbiota, and significant differences in serum metabolite levels. After treatment with YNKB for 12 weeks, serum lipid profiles and disrupted gut microbiota structure were shown to be regulated. Oral administration of high-doses of YNKB resulted in significant lipid-lowering activity against hyperlipidemia in apoE-deficient mice, which might be associated with the composition alterations of the gut microbiota and changes in serum metabolite levels. These findings highlight that YNKB, as a medicine-food formulation derived from Sichuan dark tea, can prevent dyslipidemia and research can improve the understanding of its mechanisms and the pharmacological rationale for its preventive use.

## Supporting information

S1 Table The sixteen traditional Chinese herbs of YNKB. (DOC)

S2 Table Composition of normal and high-fat diets (HFD). (DOCX)

S1 Fig The HPLC-MS analysis of NYKB. (DOCX)

## Abbreviations

HFD: High-fat diet
TG: Triglyceride
TC: Total cholesterol
LDL-c: Low-density lipoprotein cholesterol
HDL-c: High-density lipoprotein cholesterol
TCM: Traditional Chinese medicine
CFDA: China Food and Drug Administration
AST: Aspartate transaminase
ALT: Alanine transaminase
OTUs: Operational taxonomic units
PcoA: Principal coordinates analysis
