# Supplementary material for: Microbial and metabolomic remodeling by a formula of Sichuan dark tea improves hyperlipidemia in apoE-deficient mice
Source: PLoS One. 2019 Jul 3;14(7):e0219010. doi: 10.1371/journal.pone.0219010 (PMC6608967; doi:10.1371/journal.pone.0219010)
Supplement: S1 Table — (DOC) [file pone.0219010.s001.doc]

Supplementary table 1. The sixteen traditional Chinese herbs of YNKB.

| Common Chinese name | Common name | Specimen number | Latin name |
| --- | --- | --- | --- |
| Heye | Nelumbinis folium | No. 275 | *Nelumbo nucifera Gaertn*. |
| Shanzha | Crataegi Fructus | No. 31 | *Crataegus pinnatifida Bge.* |
| Gouqizi | Lycii Fructus | No. 249 | *Lycium barbarum L.* |
| Sangye | Mori Folium | No. 297 | *Morus alba L.* |
| Shanyao | Dioscoreae Rhizoma | No.28 | *Dioscorea opposita Thunb.* |
| Baihe | Lily Bulbus | No.132 | *Lilium lancifolium Thunb* |
| Fuling | Poria | No.240 | *Poria cocos (Schw.) Wolf* |
| Gancao | Glycyrrhizae Radix ET Rhizoma | No.86 | *Glycyrrhiza uralensis Fisch* |
| Gaoliangjiang | Alpiniae Officinarum Rhizoma | No. 287 | *Alpinia officinarum Hance* |
| Gegen | Puerariae Lobatae Radix | No.333 | *Pueraria lobata (Willd.) Ohwi* |
| Jiegeng | Platycodon Radix | No.277 | *Platycodon grandiflorum (jacp.) A. DC.* |
| Lianzi | Nelumbinis Semen | No.273 | *Nelumbo nucifera Gaertn*. |
| Machixian | Portulacae Herba | No.49 | *Portulaca oleracea L.* |
| Rougui | Cinnamomi Cortex | No.136 | *Cinnamomum cassia Presl* |
| Yuzhu | Polygonati Odorati Rhizoma | No.84 | *Polygonatum odoratum (MilL.) Druce* |
| Zhizi | Gardeniae Fructus | No.248 | *Gardenia jasminoides Ellis* |
