# Supplementary material for: Microbial and metabolomic remodeling by a formula of Sichuan dark tea improves hyperlipidemia in apoE-deficient mice
Source: PLoS One. 2019 Jul 3;14(7):e0219010. doi: 10.1371/journal.pone.0219010 (PMC6608967; doi:10.1371/journal.pone.0219010)
Supplement: S2 Table — (DOCX) [file pone.0219010.s002.docx]

Supplementary table 2. Composition of normal and high-fat diets (HFD).

| Component | Normal diet | HFD |
| --- | --- | --- |
| Casein, 30 Mesh(g) | 200 | 200 |
| L-Cystine(g) | 3 | 3 |
| Corn Starch(g) | 315 | 72.8 |
| Maltodextrin 10(g) | 35 | 100 |
| Sucrose(g) | 350 | 172.8 |
| Cellulose, BW200(g) | 50 | 50 |
| Soybean Oil(g) | 25 | 25 |
| Lard(g) | 20 | 177.5 |
| Mineral Mix S10026(g) | 10 | 10 |
| DiCalcium Phosphate(g) | 13 | 13 |
| Calcium Carbonate(g) | 5.5 | 5.5 |
| Potassium Citrate, 1 H2O(g) | 16.5 | 16.5 |
| Vitamin Mix V10001(g) | 10 | 10 |
| Choline Bitartrate(g) | 2 | 2 |
| FD&C Yellow Dye | #5 | #40 |
