# Supplementary material for: Microbial and metabolomic remodeling by a formula of Sichuan dark tea improves hyperlipidemia in apoE-deficient mice
Source: PLoS One. 2019 Jul 3;14(7):e0219010. doi: 10.1371/journal.pone.0219010 (PMC6608967; doi:10.1371/journal.pone.0219010)
Supplement: S1 Fig — (DOCX) [file pone.0219010.s003.docx]

**Supplementary Figure 1. The HPLC-MS analysis of NYKB**

M=174

Mass Calc. Mass mDa PPM DBE i-FIT Formula

175.1158 175.1123 3.5 20.0 5.5 8.6 C12H15O

175.1099 5.9 33.7 2.5 7.6 C10H16ONa

M=194

Mass Calc. Mass mDa PPM DBE i-FIT Formula

195.0882 195.0869 1.3 6.7 0.5 11.9 C7H15O6

195.0810 7.2 36.9 9.5 21.7 C14H11O

195.0786 9.6 49.2 6.5 21.5 C12H12O Na

195.0997 -11.5 -58.9 1.5 24.0 C9 H16 O3Na

195.1021 -13.9 -71.2 4.5 24.1 C11H15O3

M=256

Mass Calc. Mass mDa PPM DBE i-FIT Formula

257.1428 257.1389 3.9 15.2 3.5 52.9 C13H21O5

257.1365 6.3 24.5 0.5 54.6 C11H22O5Na

257.1517 -8.9 -34.6 4.5 20.6 C15H22O2Na

257.1330 9.8 38.1 12.5 45.7 C20H17

257.1542 -11.4 -44.3 7.5 20.2 C17H21O2

M=306

Mass Calc. Mass mDa PPM DBE i-FIT Formula

307.0907 307.0970 -6.3 -20.5 12.5 87.0 C19H15O4

307.0818 8.9 29.0 8.5 96.1 C15H15O7

307.1005 -9.8 -31.9 0.5 111.5 C10H20O9Na

307.0794 11.3 36.8 5.5 103.3 C13H16O7Na

307.0946 -3.9 -12.7 9.5 91.9 C17H16O4Na

M=547

Mass Calc. Mass mDa PPM DBE i-FIT Formula

570.1904 570.1893 1.1 1.9 20.5 5623.2 C34H29NO6Na

570.1917 -1.3 -2.3 23.5 5583.0 C36H28NO6

570.1858 4.6 8.1 32.5 5621.1 C43H24NO

570.1834 7.0 12.3 29.5 5662.9 C41H25NONa

570.1764 14.0 24.6 19.5 5804.4 C32H28NO9

M=478

Mass Calc. Mass mDa PPM DBE i-FIT Formula

479.2387 479.2375 1.2 2.5 21.5 2773093.0 C36H31O

479.2340 4.7 9.8 -0.5 2773065.8 C18H39O14

479.2434 -4.7 -9.8 12.5 2773083.3 C29H35O6

479.2492 -10.5 -21.9 3.5 2773073.5 C22H39O11

479.2281 10.6 22.1 8.5 2773074.8 C25H35O9

M=772

Mass Calc. Mass mDa PPM DBE i-FIT Formula

795.2094 795.2054 4.0 5.0 26.5 92.0 C44H36O13Na

795.2089 0.5 0.6 48.5 91.8 C62H28Na

795.2112 -1.8 -2.3 17.5 102.8 C37H40O18Na

795.2078 1.6 2.0 29.5 88.2 C46H35O13

795.2136 -4.2 -5.3 20.5 96.8 C39H39O18

M=822

Mass Calc. Mass mDa PPM DBE i-FIT Formula

845.2451 845.2457 -0.6 -0.7 46.5 33.2 C63H34O2Na

845.2445 0.6 0.7 27.5 47.1 C47H41O15

845.2480 -2.9 -3.4 15.5 59.7 C38H46O20Na

845.2421 3.0 3.5 24.5 50.0 C45H42O15Na

845.2481 -3.0 -3.5 49.5 31.5 C65H33O2
